# AI-Driven Innovations in Transfusion Medicine: A Narrative Synthesis of Current Reviews

**DOI:** 10.3390/medsci14010010

**Published:** 2025-12-25

**Authors:** Daniele Giansanti, Claudia Cosenza

**Affiliations:** 1 Centro Nazionale Intelligenza Artificiale e Tecnologie Innovative per la Salute, Istituto Superiore di Sanità, Via Regina Elena 299, 00161 Rome, Italy; 2 Facoltà di Medicina e Psicologia, Università Sapienza, Ospedale S. Andrea, Via di Grottarossa 1035, 00189 Rome, Italy; claudiacosenza02@libero.it

**Keywords:** blood transfusion, transfusion medicine, artificial intelligence

## Abstract

**Background:** Recent advancements in blood transfusion and transfusion medicine have increasingly integrated innovative technologies, including artificial intelligence (AI), machine learning, and computational intelligence. Despite numerous reviews on these topics, a comprehensive synthesis of the existing evidence is lacking. **Objective:** This narrative review of reviews aims to summarize and critically appraise the current literature on AI-driven and emerging technological approaches in blood transfusion, providing a structured overview for researchers and clinicians. **Methods:** A total of 19 reviews were selected through a systematic search strategy. Studies were assessed for methodological quality, scope, and clinical relevance, using adapted criteria from narrative review checklists. Data were extracted regarding the type of technology, application in transfusion medicine, study population, and reported outcomes. **Results:** The included reviews highlight several key domains: AI-assisted prediction of transfusion requirements, automated blood typing and crossmatching, advanced monitoring of blood products, and integration of computational models in blood banking workflows. Most studies reported promising applications but revealed substantial heterogeneity in methods, limited clinical validation, and variable reporting quality. **Conclusions:** AI and emerging technologies offer significant potential to improve the safety, efficiency, and personalization of blood transfusion. However, standardization of study designs, comprehensive validation, and robust reporting are essential to translate these innovations into routine clinical practice. This review of reviews provides a structured synthesis to guide future research and implementation strategies in transfusion medicine.

## 1. Introduction

### 1.1. From Early Experiments to Modern Transfusion Practices

Blood transfusion has a long and fascinating history, marked by bold experimentation and progressive scientific breakthroughs. Early attempts to transfuse blood between humans were documented as far back as the 17th century, but these interventions were largely anecdotal and often fatal [1].

A decisive milestone was the discovery of the ABO blood group system by Karl Landsteiner in 1901, which provided the first scientific explanation for transfusion reactions and enabled safe, compatible transfusions [2]. This was followed by the identification of other clinically relevant blood groups, such as the Rh system, which further reduced immunologic complications [3].

Despite these advances, the practical use of transfusion was limited by coagulation. The introduction of anticoagulants, especially sodium citrate, enabled the storage of blood and led to the first organized blood banks. Richard Weil’s early work in 1915 demonstrated the feasibility of citrated blood transfusions [4].

The mid-20th century saw further innovations: the development of plastic blood bags, refrigeration, and blood component separation (red cells, plasma, platelets) allowed transfusion practices to become more efficient, flexible, and safer [5]. In addition, crossmatching techniques evolved to prevent immunological incompatibility and hemolytic reactions [6].

The creation of national and international blood transfusion services in the 1940s–1960s standardized practices, improved donor recruitment, and enabled widespread distribution of blood products [1]. Key figures such as Tibor J. Greenwalt advanced the field through contributions to blood banking organization, leukocyte reduction, and quality control measures [7].

Despite remarkable progress, transfusion medicine remained a field of continuous learning, with ongoing attention to safety, immunology, storage lesions, and logistical challenges. The historical trajectory from hazardous early experiments to the modern, highly regulated practice illustrates the co-evolution of medicine, science, and technology in ensuring safe and effective transfusion therapy [8].

### 1.2. Technological Evolution in Transfusion Medicine: From Automation to AI-Driven Innovation

Building on the long history of transfusion medicine [1], the field has witnessed a profound shift powered by technological advancements. As early as the 1980s and 1990s, many blood banking laboratories began adopting automation systems for tasks such as blood typing, crossmatching, and serology. These automated platforms increased throughput, reduced human error, and improved traceability of testing processes [9]. More advanced immunohematology techniques—such as column agglutination and solid-phase assays—became compatible with semi- and fully automated instruments, further improving the objectivity and reproducibility of blood bank testing [10].

The integration of computerized blood banking software soon followed, enabling seamless management of donor records, inventory control, and quality assurance. Such digital systems afforded better traceability, enabled real-time decision-making, and reduced logistical inefficiencies within blood centers.

In more recent years, artificial intelligence (AI) has emerged as a transformational force in transfusion medicine. Machine learning models are being developed to predict transfusion requirements, leveraging patient data to guide orders and minimize waste [11]. AI-driven systems also support patient blood management (PBM) by assessing bleeding risk, optimizing lab test timing, and creating personalized transfusion strategies [12].

Moreover, AI is being deployed for blood inventory forecasting: predictive algorithms analyze historical usage, donation patterns, and demographic trends to optimize supply chains and reduce product expiry [13]. Early applications in hemovigilance are also promising: AI tools may detect subtle signs of transfusion reactions in real time, enhancing patient safety.

However, the transition to AI-based workflows is not without challenges. Key issues include model interpretability, data privacy, clinical validation, and integration into existing clinical infrastructure [14]. Addressing these barriers will be essential to fully harness AI’s potential in transfusion medicine.

### 1.3. Purpose

Building on the historical and technological developments described above, it is therefore important to analyze both the evolution of technological integration in transfusion medicine and the ongoing implications and challenges of artificial intelligence (AI) applications. An overview of the published reviews allows us to present these developments in a comprehensive manner.

The general aim of this study is, therefore, to provide a narrative review of reviews (NRR) with the purpose of analyzing the state of technological innovation and AI integration in transfusion medicine, with the following specific objectives:

- *Analyze overall bibliometric trends in the field:* This study aims to provide a comprehensive bibliometric overview of research output, focusing on trends and developments over time in transfusion technology, automation, and AI applications.
- *Identify established themes and categories:* Identify key areas of focus in reviews, such as automation in blood banking, predictive models for transfusion needs, patient blood management, inventory optimization, and AI-driven decision support systems.
- *Examine opportunities and challenges:* Explore the potential benefits and limitations of AI integration in transfusion medicine, including improving transfusion safety, optimizing resource allocation, enhancing patient outcomes, and addressing issues such as data privacy, algorithm interpretability, regulatory requirements, and barriers to clinical adoption.

## 2. Narrative Review Approach

### 2.1. Study Selection

This review focused exclusively on peer reviewed review articles, including narrative reviews, systematic reviews, scoping reviews, and other review studies. The rationale for restricting the analysis to secondary literature was to obtain high-level summaries of broad research domains, including well-established trends, recurring methodological approaches, and cross-cutting challenges in the application of AI to transfusion workflows.

Primary studies, algorithm-focused engineering papers without clear clinical context, and narrowly scoped technical reports were excluded, unless integrated within a selected review.

### 2.2. Assessment of Review Quality

Although narrative reviews do not follow the rigid protocols of systematic reviews, methodological rigor was ensured by following the narrative review checklist ANDJ recommended in [15]. This framework supported structured reporting across key domains, including clarity of objectives, rationale for study selection, transparency of the search strategy, analytical coherence, and critical appraisal of the included evidence.

In addition, the evaluation of technological maturity, methodological soundness, and clinical applicability was guided by the qualification criteria described in [16], which provide a structured approach for evaluating methodology based on the assessment of selected quality parameters. Each review was evaluated according to six quality parameters (N1–N6): N1, clear rationale in the introduction; N2, adequate research design; N3, clearly described methodology; N4, well-presented results; N5, conclusions justified by the results; and N6, disclosure of conflicts of interest. Parameters N1–N5 were scored on a 1-to-5 scale reflecting increasing quality, while N6 was assessed using a binary Yes/No measure. These criteria were applied qualitatively, ensuring alignment between technological capabilities, clinical relevance, and real-world feasibility.

### 2.3. Data Extraction

A structured literature search was performed across three major bibliographic databases: PubMed, Scopus, and Web of Science (WoS). These databases were selected to capture a broad spectrum of clinically and medically relevant literature, including reviews addressing AI applications in transfusion medicine, operational workflows, and patient care outcomes. The composite search key used for querying the databases is reported in Box 1, ensuring comprehensive retrieval of relevant reviews.

Box 1 **Used composite key.**

*((hematology[Title/Abstract] OR “blood analysis”[Title/Abstract] OR “blood diagnostics”[Title/Abstract] OR “blood sample”[Title/Abstract] OR “blood transfusion”[Title/Abstract] OR “transfusion medicine”[Title/Abstract] OR “blood banking”[Title/Abstract])*

*AND*

*(“artificial intelligence”[Title/Abstract] OR “machine learning”[Title/Abstract] OR “deep learning”[Title/Abstract] OR “artificial neural network”[Title/Abstract] OR “computational intelligence”[Title/Abstract]))*

Data extraction focused exclusively on clinically and medically relevant aspects of AI applications in transfusion medicine. Studies that were purely mathematical, computational, or focused on algorithmic performance without direct implications for patient care, clinical workflows, or operational outcomes were explicitly excluded. For studies emphasizing clinically and operationally relevant AI applications in transfusion medicine, the following elements were focuses:

- Type of technology or AI approach, emphasizing applications with potential clinical impact.
- Specific application within transfusion medicine, including transfusion decision support, product safety, diagnostics, and operational workflow optimization.
- Clinical or operational context, encompassing patient care settings and blood bank operations, with a focus on outcomes affecting safety, efficiency, or quality of care.
- Study population or healthcare setting, when reported, to assess applicability across different patient groups or institutional environments.
- Reported outcomes, including clinical, safety, or operational indicators; purely computational performance metrics were disregarded unless linked to tangible clinical benefit.
- Methodological limitations and research gaps, highlighting recurring themes, consolidation of knowledge, and areas requiring further validation.

Priority was given to recent reviews and those that informed prior syntheses, ensuring coverage of well-established themes and emerging trends in the medical literature. This approach supports a clinically oriented narrative synthesis, emphasizing the integration of AI in transfusion medicine while avoiding overrepresentation of studies without patient-centered relevance.

Given the heterogeneity among reviews—in conceptual scope, methodological depth, level of clinical validation, and reporting standards—a thematic synthesis approach was adopted. The extracted information was grouped into emergent domains such as AI-assisted prediction of transfusion needs, automated blood typing and compatibility testing, monitoring of blood products, process optimization in blood banks, and integration of computational intelligence in transfusion decision-making.

### 2.4. Rationale for a Narrative Synthesis

Unlike systematic reviews, which impose strict inclusion criteria and emphasize quantitative aggregation, the narrative approach allowed exploration of conceptual evolution, technological trajectories, and persisting research gaps. This flexibility was essential due to the wide variation in the maturity of AI applications, the rapidly evolving technological landscape, and the diverse clinical and operational settings in which these technologies are deployed.

By integrating insights from existing reviews, this study provides a structured, high-level synthesis that highlights consolidated knowledge, identifies methodological and implementation challenges, and outlines promising directions for future research and clinical translation in AI-enabled transfusion medicine.

## 3. Synthesis of Evidence

### 3.1. Bibliometric Trends: A Narrative Comparison

We carried out two bibliometric searches on PubMed to understand how scientific attention has evolved around blood transfusion, both in its traditional form and in combination with artificial intelligence (AI). These two searches were performed using two distinct query strategies:

- Key 1 from Box 2, designed to capture studies explicitly connecting *blood transfusion* and *AI*;
- Key 2 from Box 2, focused solely on *blood transfusion* without any reference to AI.

This approach allowed us to compare the evolution of two related but fundamentally different research landscapes. What emerges is the contrast between a long-established field, supported by over a century of literature, and a much younger domain that has expanded almost entirely within the last decade.

Box 2 **Used composite key for the Pubmed search.**

*((“blood transfusion”[Title/Abstract] OR “transfusion medicine”[Title/Abstract] OR “blood banking”[Title/Abstract])*

*AND*

*(“artificial intelligence”[Title/Abstract] OR “machine learning”[Title/Abstract] OR “deep learning”[Title/Abstract] OR “artificial neural network”[Title/Abstract] OR “computational intelligence”[Title/Abstract]))*

*--------*

*((“blood transfusion”[Title/Abstract] OR “transfusion medicine”[Title/Abstract] OR “blood banking”[Title/Abstract]))*

#### 3.1.1. Blood Transfusion & AI: A Rapidly Emerging Field

The first search—conducted with Key 1 of Box 2, which combines blood transfusion–related terms with AI, machine learning, and computational approaches—identified 205 studies published since 1998. Of these, 24 were reviews, representing 11.7% of the total. One of the most striking observations is the temporal concentration of this literature.

- 199 studies (97.1%) were published in the last 10 years.
- 181 studies (88.3%) were published in the last 5 years.

When we look at the distribution within the last decade, we find that 91.0% of all publications in the past ten years occurred in just the most recent five-year window. This extremely compressed timeline underscores how the intersection between AI and transfusion medicine is a recent but rapidly accelerating area of scientific interest. The surge is likely driven by recent advances in machine learning, automated data interpretation, decision-support tools, and the wider movement toward precision medicine.

#### 3.1.2. Blood Transfusion Alone: A Historically Rich and Stable Field

The second search—conducted with Key 2 of Box 2, deliberately excluding AI-related terms—reveals a completely different historical pattern. The earliest indexed article dates back to 1874, describing a case of direct arterial blood transfusion from animals, marking the beginning of a long scientific tradition [17].

From that initial publication to today, the field has produced 55,904 studies, including 6395 reviews—about 11.4% of the total, a proportion remarkably similar to that of the AI subset.

Despite covering more than 150 years of research, the field continues to show steady growth:

- 21,405 studies (38.3%) were published in the last 10 years;
- 12,014 studies (21.5%) in the last 5 years.

Within these recent years, 56.1% of the decade’s publications occurred in the last five years. This shows a sustained but non-explosive expansion—typical of a mature field where growth reflects ongoing refinement rather than abrupt technological disruption.

#### 3.1.3. Comparative Interpretation: Two Speeds of Scientific Evolution

When we place the results of the two searches side by side—as summarized in Table 1—the difference in scale, pace, and scientific maturity becomes immediately evident.

The field of blood transfusion alone represents a deep and historically grounded discipline, supported by more than 55,000 publications accumulated over a century and a half. Its growth pattern is steady and cumulative, reflecting continuous clinical refinement and incremental scientific progress.

In contrast, the literature at the intersection of blood transfusion and AI is not only much smaller—about 273 times smaller—but also extraordinarily recent. The fact that nearly all publications (over 97%) have appeared in the last decade, and most of those (88%) in the last five years, indicates that this research area is experiencing an intense phase of expansion. The 91% concentration of decade-specific publications in the last five years further confirms that we are witnessing a field undergoing rapid acceleration.

This comparison highlights two complementary realities:

1. Blood transfusion remains a robust, historically rich discipline with a stable publication trajectory.
2. AI-driven transfusion research is reshaping the field from the inside, introducing new computational paradigms and methodological opportunities that are changing how transfusion medicine is conceptualized, analyzed, and practiced.

In short, traditional transfusion medicine provides the long-standing clinical knowledge, while AI introduces a new strategic acceleration that is redefining research priorities and clinical expectations. Together, they outline a field undergoing transformation at two speeds: one historical and continuous, the other rapid and technologically driven.

### 3.2. Common Messages and Themes

#### 3.2.1. Common Message

A total of 19 review articles met eligibility criteria and were included for full analysis [18,19,20,21,22,23,24,25,26,27,28,29,30,31,32,33,34,35,36].

Based on the 19 selected review studies, the evidence conveys a clear and consistent message: the integration of artificial intelligence across critical care, transfusion medicine, and perioperative settings is shifting the focus from reactive treatment to proactive predictive and decision-support capabilities, although challenges remain regarding data quality, model heterogeneity, and limited clinical integration.

In perioperative and intensive care contexts, machine learning models demonstrate the ability to anticipate critical events such as intraoperative hypotension [23], the need for blood transfusion [21,30], ventilator-associated pneumonia [33], and complex scenarios like extracorporeal membrane oxygenation (ECMO) management [18]. Even in high-demand settings such as major trauma [30], battlefield care, and resource-limited environments [22], AI is proposed as a tool to standardize rapid decisions and reduce clinical variability.

Within transfusion medicine, three converging lines of application emerge:

1. Optimization of component quality—from red blood cells [35,36] and platelets [29] to advanced deep learning–driven quality control systems [26];
2. Demand prediction and supply management—through forecasting models leveraging big data and advanced analytics [34];
3. Enhancement of safety and clinical support—including predictive models for adverse transfusion reactions [20], donor management systems [19], and broader reviews outlining opportunities, challenges, and ethical considerations [25].

Additional applications—from predicting postoperative delirium in older adults [24] to outcomes in out-of-hospital cardiac arrest [31]—reinforce the same trend: AI does not replace clinical judgment but extends the informational horizon, detects early warning signals, and supports complex real-time decision-making.

Emerging approaches based on federated learning [27] provide a practical path to overcome one of the main barriers in the field: fragmented data, which limits model generalizability and robustness.

In summary, the 19 reviews converge on a key point: digital transformation driven by AI in transfusion medicine and critical care is underway, but full potential requires infrastructure, governance, multicenter validation, and seamless clinical integration.

#### 3.2.2. Emerging Themes

Recent literature underscores the growing role of artificial intelligence (AI) in transfusion medicine, particularly in enhancing clinical decision-making, optimizing donor management, and improving transfusion safety [18,19,20,21,22,23,24,25,26,27,28,29,30,31,32,33,34,35,36]. Sharabi Goldenberg et al. (2025) describe a case of a patient on veno-arterial extracorporeal membrane oxygenation (VA-ECMO) in which AI, specifically a large language model, was employed to support rapid literature synthesis and clinical decision-making regarding blood transfusion. While the AI tool facilitated information retrieval and highlighted relevant clinical considerations, the authors emphasized that ultimate responsibility for transfusion decisions remains with the human clinician, underscoring AI’s role as an augmentative rather than substitutive tool in critical care settings [18].

Beyond individual patient support, AI is increasingly applied to blood donor management. Badawi (2025) reviews how AI techniques, including machine learning, natural language processing, and robotic process automation, can streamline donor recruitment, retention, and engagement. By analyzing large datasets to predict donation patterns, forecast blood demand, and identify rare donors, AI enables more dynamic and efficient management of blood resources. The integration of AI-driven chatbots further facilitates donor communication, enhancing retention and operational efficiency. Nonetheless, the author notes that successful implementation requires careful attention to data quality, ethical considerations, infrastructure, technical expertise, and cybersecurity [19].

In the domain of transfusion safety, ShojaeiBaghini et al. (2025) systematically examined AI applications for identifying and predicting adverse transfusion reactions (ATRs). Their review found that AI models, particularly Random Forest algorithms, were effective in evaluating transfusion risks, outcomes, and volumes, as well as classifying ATRs. However, the study highlighted significant gaps, including the lack of AI-based active management systems and the limited focus on vulnerable populations, such as pediatric patients. The authors advocate for integrating AI-driven clinical decision support systems (CDSS) with electronic health records (EHR) and personalized medicine approaches while maintaining ethical standards and patient privacy [20].

Similarly, Chen et al. (2025) assessed clinical prediction models for postoperative blood transfusion following total knee arthroplasty, incorporating both logistic regression and machine learning approaches. Key predictors included preoperative hemoglobin, age, body mass index, surgery duration, and tranexamic acid usage. The pooled analysis demonstrated moderate to excellent predictive discrimination; however, methodological limitations and a lack of external multicenter validation were noted. This underscores the need for rigorously developed AI models to ensure clinical applicability and reliable transfusion planning [21].

Finally, the application of AI extends to resource-limited and high-demand environments. Jarrassier et al. (2025) explored intensive care innovations from modern warfare, identifying technologies and strategies potentially transposable to transfusion management in low-resource settings. AI-supported monitoring, portable blood transfusion platforms, and teleconsultation/telementoring approaches were highlighted as enabling rapid, evidence-based decision-making in challenging contexts. The authors stress that successful adaptation requires validation, contextualization, and structured training programs to integrate AI effectively into clinical workflows [22].

Building on this foundation, further studies highlight AI’s role in predictive modeling and quality optimization. Koh et al. (2025) describe machine learning applications for anticipating intraoperative hypotension—a factor closely linked to blood transfusion requirements—with predictive algorithms identifying events up to 15 min in advance [23]. Wróbel et al. (2025) demonstrate AI’s potential in assessing postoperative delirium risk in elderly patients, integrating diverse physiological and neuromonitoring parameters into predictive models to enable earlier interventions [24]. Cohen et al. (2025) provide a broad overview of AI applications in transfusion medicine, from donor management and transfusion safety to resource allocation, while emphasizing workflow integration, data privacy, and equity challenges [25]. Pereira et al. (2025) focus on AI-driven quality control in blood component manufacturing, highlighting real-time monitoring, predictive analytics, and proactive error detection to reduce clinical risks [26]. Li et al. (2025) explore federated learning as a privacy-preserving approach to train AI models across multiple datasets, optimizing transfusion demand prediction, personalized treatment, and logistics without centralizing sensitive data [27].

Recent research continues to underscore the expanding role of AI in surgical and trauma settings. Duranteau et al. (2024) conducted a scoping review of machine learning models predicting transfusion requirements during surgery, using both biological and clinical variables. Logistic regression was the most commonly applied method, although other machine learning techniques were also used [28]. Trochanowska-Pauk et al. (2024) describe AI-driven analyses to optimize platelet storage, predict quality, and guide production strategies [29]. Oakley et al. (2024) reviewed machine learning models for trauma-related transfusions, highlighting excellent discrimination in some externally validated models [30]. Plodr and Chalusova (2024) emphasize AI-facilitated pre-hospital decision support for cardiac arrest patients [31], while Angthong et al. (2023) demonstrate AI’s utility in polytrauma management, particularly for hemorrhagic shock [32].

Finally, studies on transfusion management in critical care and supply logistics further illustrate AI’s potential. Frondelius et al. (2024) systematically reviewed ML-based prediction models for ventilator-associated pneumonia (VAP), highlighting risk factors such as mechanical ventilation duration and transfusions, and emphasizing the need for dynamic, time-dependent predictive models [33]. Li et al. (2023) evaluated AI-driven approaches for blood supply forecasting and inventory optimization, noting limitations in generalizability and ethical considerations [34]. Advances in omics technologies, discussed by D’Alessandro (2023), enable ML strategies to improve red blood cell quality, predict storage behavior, and personalize donor–recipient matching [35]. Lopes et al. (2023) further underline AI’s role in quality control of red blood cell units, emphasizing the need for clinical validation [36].

Collectively, these studies [18,19,20,21,22,23,24,25,26,27,28,29,30,31,32,33,34,35,36] illustrate a trajectory in which AI and ML progressively enhance multiple facets of transfusion medicine—from clinical decision support, risk prediction, and donor–recipient matching to blood product quality control and supply management—while emphasizing the importance of methodological rigor, dynamic modeling, and ethical considerations for safe integration into clinical practice. Table 2 reports a sketch of the Overviewed studies.

### 3.3. Emerging Opportunities and Challenges

Artificial intelligence (AI) is rapidly emerging as a transformative tool in transfusion medicine, offering novel approaches to anticipate transfusion needs, enhance patient safety, and optimize the management of blood resources. By leveraging large datasets, predictive algorithms, and advanced modeling techniques, AI has the potential to support clinicians in decision-making, streamline operations, and enable precision transfusion practices. However, its integration also poses important challenges related to data quality, model interpretability, regulatory compliance, and ethical considerations. The following sections detail the key opportunities and challenges informed by recent literature [18,19,20,21,22,23,24,25,26,27,28,29,30,31,32,33,34,35,36].

#### 3.3.1. Opportunities

Among the opportunities the following ones were detected in the overviewed studies:

- Predictive modeling and risk assessment: Machine learning and AI algorithms can anticipate intraoperative hypotension, postoperative delirium, and transfusion requirements in surgical and trauma patients, enabling proactive interventions to reduce complications and optimize transfusion volumes [23,24,28,30,32].
- Transfusion safety and hemovigilance: AI supports identification and prediction of adverse transfusion reactions (ATRs) and guides evidence-based risk management, enhancing patient safety, particularly in vulnerable populations such as pediatrics [20,29,36].
- Donor management and resource optimization: Advanced AI techniques, including natural language processing, robotic process automation, and federated learning, facilitate donor recruitment, retention, and rare donor identification, while optimizing blood demand forecasting and inventory management across institutions [19,25,27,34].
- Blood product quality control: Integration of AI with omics data and big data analytics allows monitoring of red blood cell and platelet quality, predicting storage stability, and informing development of novel additives, ultimately enabling precision transfusion medicine [35,36].
- Real-time clinical decision support: AI-driven systems, such as large language models and decision support tools, can synthesize literature rapidly and provide context-specific recommendations during critical care or complex interventions like VA-ECMO, acting as augmentative tools for clinicians [18,22].

#### 3.3.2. Challenges

Despite these promising opportunities, several challenges limit the safe and effective integration of AI in transfusion medicine were detected in the studies:

- Data quality, heterogeneity, and accessibility: AI models require high-quality, standardized, and interoperable datasets from electronic health records, laboratory results, and omics analyses; missing or biased data can impair predictive accuracy and generalizability [21,28,33,34].
- Algorithm interpretability and clinical trust: Complex models (deep learning or ensemble methods) may lack transparency, limiting clinician confidence and adoption; explainable AI is critical for actionable insights in high-stakes transfusion decisions [20,21,30].
- Validation and regulatory requirements: Many AI models have only internal or retrospective validation, with limited external, multicenter, or prospective studies; regulatory frameworks for AI deployment in transfusion medicine remain evolving [21,26,30,33].
- Ethical and privacy considerations: Federated learning and other privacy-preserving approaches mitigate some risks, but AI implementation must ensure patient confidentiality, equitable access, and ethical resource allocation [19,27].
- Integration into clinical workflows and training: Effective AI adoption requires seamless integration with existing electronic health records, robust IT infrastructure, and training of healthcare teams to interpret outputs and act safely [22,26,36].

Together, these opportunities and challenges illustrate a landscape in which AI has the potential to transform transfusion medicine—improving safety, personalizing treatment, and optimizing resources—while emphasizing that careful validation, ethical oversight, and clinician engagement remain essential for meaningful clinical impact.

## 4. Discussion

### 4.1. Summary, Highlights, and Recommendations

The narrative of the NNR, through the analysis of recent reviews [18,19,20,21,22,23,24,25,26,27,28,29,30,31,32,33,34,35,36], provides a comprehensive synthesis of AI applications in transfusion medicine, moving beyond the mere aggregation of individual studies. A key strength of this approach lies in its ability to identify consolidated themes—recurring patterns and issues consistently observed across multiple primary investigations. By distinguishing between isolated findings and well-established knowledge, this methodology offers a robust and reliable overview of the opportunities, challenges, and best practices for integrating AI into both clinical and operational transfusion workflows.

The NRR clearly highlights several stabilized themes:

1. Predictive modeling for transfusion needs: AI- and machine learning-based predictive models, applied in surgical procedures, trauma care, and intensive care units (ICUs), enable anticipation of transfusion requirements, optimizing timing and volume [23,28,30,32].
2. Quality control and precision transfusion: Integration of omics data with AI supports personalized donor–recipient matching, assessment of blood product quality, and prediction of storage stability [35,36].
3. Donor management and operational efficiency: AI facilitates donor recruitment, retention, and dynamic inventory management, improving logistic efficiency and demand planning [19,25,27,34].
4. Decision support in critical care: AI systems support rapid decision-making in complex scenarios, such as patients on VA-ECMO or hemorrhagic shock trauma, enhancing patient safety and timeliness of interventions [18,22,23,30].
5. Methodological rigor and ethical considerations: Reviews emphasize the need for external validation, transparency of models, interpretability, and patient privacy as prerequisites for safe and responsible adoption [20,21,26,27,33,36].

Beyond consolidating these themes, the NRR provides directly and indirectly specific added values:

- Identification of knowledge gaps: Highlight underexplored areas such as vulnerable populations (pediatric patients, frail elderly) and the absence of AI-based active management systems [20,24,30].
- Guidance for clinical and operational recommendations: Suggest priorities for future research, technology integration, and development of dynamic models [27,28,33].
- Methodological synthesis: The possibility to find and compare statistical, machine learning, and deep learning approaches, providing insights into the most robust and reproducible strategies [21,28,33].
- Implementation context: Discuss adaptation in resource-limited settings, emphasizing training, infrastructure, and telemedicine as key elements [22,26].

From the identified themes, as well as the observed opportunities, open challenges, and existing gaps, it is possible to distill the Emerging Recommendations (both direct and/or indirect) reported in Table 3

In summary, the reviews not only confirm the potential of AI in transfusion medicine but also consolidate evidence, identify stabilized themes, and provide a structured set of recommendations, forming a solid foundation for future research and responsible clinical implementation.

### 4.2. Emerging Global Market Dynamics in Transfusion Medicine and Artificial Intelligence

The global transfusion medicine market is experiencing strong growth, driven by rising demand for blood products, technological innovation, and expanding healthcare infrastructure. According to a recent report, the transfusion medicine market was valued at USD 61.2 billion in 2024 and is projected to reach USD 88.8 billion by 2030, with a compound annual growth rate (CAGR) of 6.4% over this period [37].

Parallel to this, the transfusion technology segment—which includes devices, automation, and software to support blood collection, processing, and safety—is expected to surge from USD 14.4 billion in 2023 to USD 53.9 billion by 2033, growing at an estimated CAGR of 14.11% [38].

On the diagnostics side, the blood transfusion diagnostics market is forecast to expand significantly. One analysis estimates growth from approximately USD 5.28 billion in 2025 to USD 8.79 billion by 2034, with a CAGR of 5.84% [39], while another projects a CAGR of 7.7% from 2024 (USD 5.33 billion) to 2032 (USD 9.64 billion) [40].

Meanwhile, the patient blood management (PBM) market—which overlaps with AI-driven transfusion optimization strategies—is also expanding: the global PBM market was valued at USD 14.65 billion in 2024 and is projected to reach USD 25.60 billion by 2033, at a CAGR of 6.40% [41].

These market dynamics suggest a fertile environment for AI integration. As transfusion services modernize, AI and digital technologies are expected to capture an increasing share of this expanding market—particularly in diagnostics, decision support, automated devices, and demand forecasting. Given the projected growth of core transfusion markets and technology segments, AI solutions embedded within these domains are well positioned to scale alongside the broader industry.

A realistic projection for AI-specific applications, based on market report data, estimates that the AI-driven transfusion decision support market reached USD 1.21 billion in 2024 and could grow to USD 6.38 billion by 2033 [42]. Additionally, the AI-integrated blood analyzers market is expected to expand significantly, from USD 2.81 billion in 2025 to approximately USD 19.35 billion by 2035, at a CAGR of 21.3% [43].

The data in Table 4 highlight a strong and diverse growth trajectory across both traditional and technology-driven segments of transfusion medicine. The global transfusion medicine market shows steady expansion from USD 61.2 billion in 2024 to USD 88.8 billion by 2030, at a CAGR of 6.4%, reflecting increasing demand for blood products and healthcare infrastructure expansion. This broad base represents significant potential for AI adoption in diagnostics, clinical decision support, and supply forecasting, with AI poised to enhance efficiency and patient outcomes in established markets.

The transfusion technology segment exhibits an exceptionally high CAGR of 14.11%, rising from USD 14.4 billion in 2023 to USD 53.9 billion in 2033. This rapid growth underscores the strategic opportunity for AI-enabled automation, real-time process monitoring, and predictive risk management in blood collection, processing, and safety operations.

In the blood transfusion diagnostics market, values increase from USD 5.28–5.33 billion to USD 8.79–9.64 billion by the early 2030s, with CAGRs ranging from 5.84% to 7.7%. The consistent growth indicates an expanding role for AI-assisted diagnostics and predictive testing, enabling more accurate and timely assessments that improve transfusion safety and optimize resource utilization.

The patient blood management (PBM) market, with a CAGR of 6.4%, demonstrates growing integration of AI-driven transfusion optimization strategies. AI applications in PBM can support individualized transfusion planning, risk stratification, and protocol adherence, potentially reducing unnecessary transfusions and improving clinical outcomes.

Notably, the AI-specific markets show the highest projected growth. The AI-driven transfusion decision support market is expected to expand from USD 1.21 billion in 2024 to USD 6.38 billion by 2033, reflecting the increasing adoption of predictive analytics and real-time clinical support tools. Meanwhile, AI-integrated blood analyzers are projected to grow from USD 2.8 billion to USD 19.3 billion over a decade, with an impressive CAGR of 21.3%. This highlights the transformative potential of combining automated analysis with AI-based quality control, predictive monitoring, and operational efficiency.

Overall, the table illustrates that AI integration is not just a supplementary innovation but a core driver of growth in transfusion medicine. Traditional market segments provide a stable foundation for AI deployment, while technology-focused and AI-specific areas are expanding at exceptional rates, indicating substantial opportunities for investment, clinical innovation, and improved patient care.

### 4.3. Overcoming Review-Level Gaps with Primary Research Findings

The recent body of literature, selectively curated for its contribution to emerging best practices in transfusion medicine and artificial intelligence applications (studies [44,45,46,47,48,49,50,51,52,53,54,55,56,57,58,59,60,61,62,63,64,65,66,67,68,69,70,71,72,73,74,75,76,77,78,79,80,81,82]), illustrates how AI is being leveraged to improve transfusion decision-making, optimize blood product management, and support clinicians in complex perioperative and critical care scenarios. Mapping these studies onto the emerging recommendations from Table 3 ER (1–10) provides a comprehensive view of how cutting-edge research is translating into actionable clinical strategies.

1. Standardize and improve data quality

High-quality, interoperable datasets are essential for the development of accurate and generalizable AI models. Several studies emphasize this principle: Deng et al. [44] and Kahveci [45] use robust multicenter datasets to predict surgical duration and intraoperative transfusion needs. Zhang et al. [72] and Li et al. [73] construct comprehensive datasets for predicting transfusions in neonates and occupational hearing loss, highlighting the need for standardized data collection to enable reproducible and clinically relevant models. Li et al. [48] and Sun et al. [52] also emphasize careful feature selection and data preprocessing for optimal model performance.

2. Prioritize model interpretability

For AI to be actionable, clinicians must understand and trust its outputs. Studies by Lee et al. [70] and Xu et al. [59] provide interpretable models for transfusion decisions and prediction of postoperative respiratory failure. Sun et al. [52] demonstrates interpretable predictions in perioperative blood transfusion for hip arthroplasty, while Agarwalla et al. [46] and Jiao et al. [49] use explainable machine learning to clarify risk factors for postoperative complications and pneumonia in elderly stroke patients.

3. Validate models externally

External validation through multicenter, prospective, or dynamic datasets is critical to confirm reproducibility. Wang et al. [54], Schwinn et al. [71], and Luo et al. [53] conduct multicenter and federated learning studies, validating transfusion prediction models across diverse populations. Weidman et al. [68] applies their trauma triage model to critical care transport scenarios, and Liang et al. [69] validates transfusion prediction models for elderly arthroplasty patients, demonstrating robustness in real-world clinical settings.

4. Ensure ethical AI use

Ethical deployment is central to AI adoption. Studies by Al-Riyami & Herjes [47] and Schwinn et al. [71] highlight privacy-preserving approaches such as federated learning and mitigation of bias, particularly in multicenter datasets. Ahmed et al. [78] and Li et al. [56] also integrate ethical considerations in predictive models for postoperative VTE and gastrointestinal bleeding, ensuring fair, responsible AI use.

5. Integrate AI into clinical workflows

Implementation of AI requires adaptation of clinical processes, training, and infrastructure. Gopal et al. [61] demonstrate real-time AI mortality prediction in trauma patients, while Maman et al. [62] and Chen et al. [81] show AI supporting routine decision-making in elective surgeries and PCI procedures. McBride et al. [63] explores the educational integration of AI in transfusion medicine, highlighting how workflow adaptation supports effective adoption.

6. Leverage AI for precision transfusion

AI enables individualized transfusion strategies, optimizing blood product utilization and donor–recipient matching. Sun et al. [52], Luo et al. [53], Duranteau et al. [66], and Li et al. [56] develop models predicting perioperative and post-transfusion outcomes. Jenwitheesuk et al. [51] applies AI to plasma quality control, while Zhang et al. [72] and Li et al. [73] leverage predictive modeling to guide individualized interventions, reducing unnecessary transfusions and improving patient safety.

7. Promote operational efficiency

AI contributes to better resource management and operational planning. Zhang et al. [72], Li et al. [73], and Zhang et al. [50] illustrate predictive modeling for inventory and resource allocation. Jenwitheesuk et al. [51] and Al-Riyami & Herjes [47] show AI enhancing donor management and blood product quality monitoring, aligning with the recommendation to improve operational efficiency.

8. Support real-time critical decision-making

In high-risk interventions, rapid AI-supported decisions can be life-saving. Xu et al. [59], Marsden et al. [76], and Sawma et al. [60] develop models for ICU, trauma, and cardiac surgery settings, enabling immediate guidance in critical contexts. Li et al. [64] and Peres et al. [57] also provide dynamic risk assessments for critically ill or surgical patients, supporting timely interventions.

9. Develop dynamic, time-dependent models

Time-aware models capture evolving patient states, crucial for ICU, perioperative, and trauma care. Peres et al. [57], Li et al. [64], and Jiao et al. [49] use dynamic models to predict outcomes such as pneumonia, renal replacement therapy, or postoperative complications. Zhang et al. [69] and Hu et al. [74] similarly employ longitudinal patient data to enhance predictive accuracy and inform adaptive clinical strategies.

10. Encourage transparency and reproducibility

Transparent methodology and reproducible AI development are key to building trust. Zhang et al. [50], Al-Riyami et al. [82], and Cognasse et al. [55] emphasize open code, standardized reporting, and clear methodological documentation. Studies such as Ahmed et al. [78] and Weidman et al. [80] further ensure reproducibility through rigorous validation and explicit reporting of model performance.

Overall these studies [44,45,46,47,48,49,50,51,52,53,54,55,56,57,58,59,60,61,62,63,64,65,66,67,68,69,70,71,72,73,74,75,76,77,78,79,80,81,82] collectively illustrate that AI is reshaping transfusion medicine, perioperative care, and critical care, supporting patient safety, optimizing resources, and enhancing decision-making. When framed in the context of the emerging recommendations from Table 3 ER (1–10), they reveal a coherent trajectory toward interpretable, validated, ethical, and operationally integrated AI tools that are both clinically impactful and research-grounded.

### 4.4. Overcoming Review-Level Gaps with Guidelines and Regulatory Documents on AI in Transfusion Medicine

Although there are still few guidelines specifically dedicated to AI in transfusion medicine, several international and national documents provide general principles that are broadly applicable to AI deployment in healthcare, including transfusion. Some are reported merely as examples, referring to specific investigations for further insights. These documents ensure safe, ethical, interpretable, and clinically effective use of AI, directly mapping to the emerging recommendations (ER) in Table 3.

*USA–FDA, Health Canada, and MHRA Good Machine Learning Practice (GMLP)*

The FDA, in collaboration with Health Canada and the UK MHRA, issued the Good Machine Learning Practice principles for medical device development [83,84].

While these principles are general for all medical AI, they are directly applicable to transfusion medicine, guiding the development of predictive models for donor–recipient matching, transfusion demand forecasting, and blood product quality.

- Emphasizes high-quality, representative datasets (ER 1: Standardize and improve data quality).
- Requires interpretable outputs to ensure clinicians can trust and act on AI predictions (ER 2) and transparent reporting (ER 10).
- Lifecycle monitoring supports dynamic, adaptive models in perioperative or transfusion-critical contexts (ER 9).

*European Union–AI Act & European Health Data Space (EHDS)*

The EU AI Act and EHDS set broad standards for high-risk AI systems in healthcare [85,86,87].

Although not transfusion-specific, these regulations apply to AI systems managing blood data, enabling predictive analytics for inventory control, donor–recipient compatibility, and operational efficiency (ER 6, ER 7).

- Mandates interoperable datasets (ER 1) and human oversight (ER 2, ER 4).
- Continuous post-market monitoring aligns with time-aware models for ICU, perioperative, or transfusion-critical contexts (ER 9).

*Italy–Centro Nazionale Sangue (CNS)*

CNS guidelines on telemedicine in transfusion services (“LG CNS 08”) [88,89] provide operational standards that are transfusion-specific, but the principles of data interoperability, workflow integration, and traceability are applicable to AI-enabled transfusion systems.

- Facilitates workflow integration of AI (ER 5) and real-time decision support in high-risk interventions (ER 8).
- Ensures data quality and reproducibility for AI model development (ER 1, ER 10).

*Other International Perspectives*

- UK (NHS AI Guidelines): Provide general AI guidance emphasizing ethics, interpretability, and workflow integration (ER 2, ER 4, ER 5), also directly relevant to transfusion AI [90,91].
- Canada (Health Canada AI Guidance): Focuses on clinical validation and post-market monitoring (ER 3, ER 9), applicable also to transfusion predictive systems [92].
- China (National Health Commission AI Guidelines): Highlight standardized data, interoperability, and dynamic monitoring (ER 1, ER 3, ER 9), also applicable in AI &transfusion workflows [93].

Overall the above documents are general AI guidance, but they can be directly applied to transfusion medicine, especially for predictive modeling, workflow integration, and ethical oversight.

They address critical ER areas: data quality (ER 1), interpretability and transparency (ER 2, ER 10), ethical use (ER 4), workflow integration (ER 5), precision transfusion (ER 6), operational efficiency (ER 7), critical decision-making (ER 8), and dynamic, time-dependent modeling (ER 9).

National and regional bodies, like CNS in Italy, further tailor these general principles to transfusion-specific operations, supporting AI deployment in blood services.

### 4.5. Limitations

Although this narrative review of reviews provides a comprehensive synthesis of current knowledge on AI in transfusion medicine, certain limitations are inherent to its design. The review is narrative and not systematic (with the related design rigor); however, precisely because of this, this approach allows for a flexible, integrative perspective that highlights overarching trends and conceptual insights. First, the narrative nature may introduce selection and interpretive bias, and the included reviews are heterogeneous in scope, quality, and focus, resulting in variable evidence that may not fully capture emerging AI technologies and their clinical integration in transfusion services. Second, there remains a relative scarcity of transfusion-specific AI guidance in the literature, as most recommendations are extrapolated from general healthcare AI reviews. Third, contextual factors such as regulatory frameworks, operational workflows, and ethical considerations are often underrepresented.

These limitations also highlight opportunities. Integrating more recent primary studies (e.g., studies [44,45,46,47,48,49,50,51,52,53,54,55,56,57,58,59,60,61,62,63,64,65,66,67,68,69,70,71,72,73,74,75,76,77,78,79,80,81,82]) allows for a more up-to-date mapping of AI applications directly in transfusion medicine. Incorporating international and national documents—such as FDA GMLP, EU AI Act, European Health Data Space, NHS AI Guidelines, Health Canada guidance, CNS telemedicine recommendations, and Chinese National Health Commission AI guidance—can address gaps related to ethical, regulatory, and operational contexts. This linkage enables alignment with the emerging recommendations (ER 1–10), supporting safer, interpretable, and clinically effective AI deployment in transfusion practice while fostering dynamic, time-dependent models and workflow integration.

## 5. Future Directions

Building on the emerging recommendations (ER, Table 3), insights from recent reviews [18,19,20,21,22,23,24,25,26,27,28,29,30,31,32,33,34,35,36], primary research studies [44,45,46,47,48,49,50,51,52,53,54,55,56,57,58,59,60,61,62,63,64,65,66,67,68,69,70,71,72,73,74,75,76,77,78,79,80,81,82], and market and regulatory analyses, several avenues for future research and clinical implementation can be identified. First, there is a pressing need to address underexplored populations, including pediatric and frail elderly patients, as well as complex perioperative and critical care scenarios, through multicenter, longitudinal studies that enable validation of dynamic, time-aware models [28,30,57,64]. Such research would strengthen model generalizability and support precision transfusion strategies tailored to evolving patient states [28,30,33].

Second, AI model development should continue to prioritize interpretability, transparency, and reproducibility, leveraging standardized, high-quality datasets [21,28,33,44], federated learning approaches [47,71], and open code practices [50,55] to ensure safe, reliable, and generalizable deployment across diverse healthcare environments. Externally validated, explainable models are crucial to foster clinician trust and enable real-time decision-making in high-risk interventions such as VA-ECMO, trauma care, or perioperative management [18,22,23,30,59].

Third, integration of AI into clinical and operational workflows remains a critical challenge. Future efforts should focus on embedding predictive tools seamlessly into transfusion services, supporting training, workflow adaptation, and infrastructure requirements, while also considering implementation in resource-limited or remote settings [22,26,61,62,63]. These strategies will be essential for translating predictive insights into improved patient outcomes and operational efficiency, including donor management, inventory control, and demand forecasting [19,25,27,34,50,51].

Fourth, ethical, legal, and regulatory considerations must be proactively addressed, ensuring compliance with the FDA/Health Canada Good Machine Learning Practice (GMLP) [83,84], EU AI Act and EHDS [85,86,87], and national guidelines such as those from CNS Italy [88,89]. Developing transfusion-specific frameworks for data governance, fairness, human oversight, and lifecycle monitoring will support responsible AI adoption while maintaining patient safety, privacy, and equity [20,21,26,27,33,36].

Fifth, technological and market opportunities indicate strong potential for scalable AI applications, including real-time clinical decision support, predictive blood product management, and automated diagnostics [37,38,39,40,41,42,43]. Continued investment, partnerships, and innovation in AI-driven transfusion technologies will be critical to capture these opportunities and to align clinical practices with evolving global market demands.

Finally, future research should evaluate the clinical and operational impact of AI-driven interventions in real-world settings, combining predictive analytics with omics and patient blood management strategies [35,36,52,56,66]. Emphasis on prospective validation, dynamic model adaptation, and post-market monitoring will help translate current knowledge into actionable, evidence-based, and clinically impactful solutions. Collectively, these directions provide a roadmap to advance AI integration in transfusion medicine responsibly, efficiently, and safely, building on the consolidated themes and recommendations identified throughout this review [18,19,20,21,22,23,24,25,26,27,28,29,30,31,32,33,34,35,36,44,45,46,47,48,49,50,51,52,53,54,55,56,57,58,59,60,61,62,63,64,65,66,67,68,69,70,71,72,73,74,75,76,77,78,79,80,81,82].

## 6. Conclusions

Artificial intelligence is emerging as a transformative tool across the transfusion chain, with consolidated evidence supporting applications in donor screening, component quality, inventory forecasting, immunohematology, and prediction of transfusion reactions. This narrative review of reviews shows that the field is advancing, but still characterized by heterogeneous methodologies, variable data quality, and uneven maturity across clinical domains.

Across the included reviews, several converging themes emerge: AI can enhance operational efficiency, support earlier and more accurate clinical decision-making, and improve traceability and safety. At the same time, limitations remain significant. Evidence is fragmented, rarely transfusion-specific, and often based on retrospective datasets or narrow use cases. Many promising models lack external validation, prospective testing, or integration into routine workflows. Yet, important opportunities are clear. By incorporating recent primary studies, international guidelines and documents, and aligning high-level insights with real-world transfusion scenarios, this review highlights a growing readiness of AI tools to complement laboratory automation, strengthen quality systems, and support time-critical decisions in complex care settings. Advancing the field now requires three priorities:

(1) More robust and transfusion-focused evidence, including prospective and multicenter studies;

(2) Better data standardization and interoperability, enabling reproducible and generalizable models; 

(3) Closer integration of AI into clinical workflows, ensuring interpretability, reliability, and sustained clinical value.

In sum, AI offers substantial potential to improve safety, efficiency, and responsiveness in transfusion medicine. Realizing this promise will depend on rigorous validation, thoughtful implementation, and a continued effort to bridge current gaps in evidence and practice.

## Figures and Tables

**Table 1:** Comparison of bibliometric trends between Blood Transfusion & AI and Blood Transfusion alone.

Metric	Blood Transfusion & AI	Blood Transfusion (No AI)	Comparative Insight
Total publications	205	55,904	BT-alone is ~273× larger
Reviews (% of total)	24 (11.7%)	6395 (11.4%)	Similar review proportion
Last 10 years	199 (97.1%)	21,405 (38.3%)	AI-related research is almost entirely recent
Last 5 years	181 (88.3%)	12,014 (21.5%)	AI subset shows sharply accelerated growth
Last 5/Last 10 years	91.0%	56.1%	AI field is highly concentrated in the most recent years

**Table 2:** Overview of Artificial Intelligence Applications in Transfusion Medicine: Key Studies, Focus Areas, and Emerging Roles.

Reference	Brief Description	Transfusion-Related Focus	Role of AI
[18] Sharabi Goldenberg et al., 2025	Case-based narrative review of a patient on VA-ECMO, illustrating the use of AI to rapidly synthesize literature and support complex transfusion-related decision-making.	Support for critical transfusion decisions in ECMO patients	Large language model provided fast literature retrieval and highlighted clinical considerations; ultimate decision remains clinician’s, AI is augmentative
[19] Badawi, 2025	Review of AI for blood donor management including ML, NLP, and robotic process automation	Donor recruitment, retention, engagement, and predicting blood demand	AI predicts donation patterns, identifies rare donors, improves operational efficiency; chatbots enhance communication; emphasizes need for ethical, secure, and high-quality data
[20] ShojaeiBaghini et al., 2025	Systematic review of AI applications for identifying ATRs	Risk prediction and classification of ATRs	Random Forest and other ML models predict transfusion risk, volume, and outcomes; highlights gaps in pediatric populations and lack of active management systems; advocates CDSS/EHR integration
[21] Chen et al., 2025	Postoperative transfusion prediction following total knee arthroplasty	Predicting individual transfusion requirements	Logistic regression and ML models integrate patient and surgical factors (Hb, age, BMI, surgery duration, tranexamic acid) to predict need; moderate-to-excellent discrimination; highlights need for multicenter validation
[22] Jarrassier et al., 2025	Adaptation of ICU innovations from modern warfare to low-resource settings	Transfusion in challenging environments	AI-assisted monitoring, portable transfusion platforms, teleconsultation; enables rapid evidence-based decisions; requires validation and structured training
[23] Koh et al., 2025	ML for predicting intraoperative hypotension, associated with transfusion risk	Perioperative transfusion planning	Predictive algorithms like Hypotension Prediction Index anticipate events up to 15 min in advance; potential to reduce transfusion volume, postoperative complications, and hospital stay
[24] Wróbel et al., 2025	ML to predict postoperative delirium in elderly surgical patients	POD-associated transfusion risk	Models integrate inflammatory, metabolic, neuromonitoring parameters to allow early preventive interventions and enhance patient safety
[25] Cohen et al., 2025	Broad overview of AI in transfusion medicine	Donor management, transfusion safety, inventory and resource allocation	ML, DL, NLP improve transfusion need prediction, hemovigilance, antigen phenotyping, and inventory control; early promise but implementation challenges remain
[26] Pereira et al., 2025	AI-driven quality control in blood component manufacturing	Manufacturing, storage, pathogen risk	Real-time monitoring, predictive analytics, proactive error detection; predicts storage stability, donor suitability, pathogen risks; improves compliance and clinical outcomes
[27] Li et al., 2025	Federated learning across multiple datasets for transfusion medicine	Multi-institutional transfusion demand, personalized planning	Privacy-preserving AI enables cross-site training without centralizing data; supports prediction, planning, and logistics while mitigating bias and governance issues
[28] Duranteau et al., 2024	Scoping review of ML models for surgical transfusion	Surgical transfusion requirements	40 studies analyzed; biological and clinical predictors used (Hb, platelets, coagulation, creatinine, age, blood loss, ASA); logistic regression most used; need for transparency, standardized methods, open code
[29] Trochanowska-Pauk et al., 2024	AI for platelet storage optimization	Platelet quality, storage lesions, contamination risk	Mathematical/statistical models predict storage outcomes, guide production, and optimize quality; supports safer and more effective platelet transfusion
[30] Oakley et al., 2024	ML models predicting blood transfusion in trauma	Trauma transfusion needs	25 models reviewed; some externally validated; supports individualized transfusion strategies; highlights importance of prospective validation and methodological rigor
[31] Plodr & Chalusova, 2024	Pre-hospital AI decision support in cardiac arrest	Hemorrhagic risk and transfusion	AI supports rapid triage, assesses bleeding risk, and informs timely transfusion interventions in time-critical scenarios
[32] Angthong et al., 2023	AI in polytrauma management	Shock, bleeding, transfusion decision-making	Eight studies analyzed; ML models show good-to-excellent performance predicting patient management; enables real-time decision support in complex trauma care
[33] Frondelius et al., 2024	ML models predicting VAP	VAP-related transfusion risk	Static models identify risk factors including transfusions; pooled AUC 0.88; highlights need for dynamic, time-dependent predictive systems for real-time use
[34] Li et al., 2023	ML, hybrid, and time-series models for blood supply management	Blood demand forecasting, inventory optimization	Data-driven approaches improve accuracy using EHR predictors; limitations include generalizability, ABO compatibility, ethics, and field-specific metrics
[35] D’Alessandro, 2023	Integration of omics (genomics, proteomics, lipidomics, metabolomics) with AI	Blood product quality, donor-recipient matching	Large datasets inform ML models to predict RBC behavior, optimize storage, develop additives, and enable precision transfusion; step toward individualized medicine
[36] Lopes et al., 2023	Big data and AI for quality control of RBC units	RBC quality monitoring	Multiple analytical strategies available; clinical implementation limited; emphasizes need for validation to ensure predictive insights translate into safer transfusion

Abbreviations: AI: Artificial Intelligence; VA-ECMO: Veno-Arterial Extracorporeal Membrane Oxygenation; ECMO: Extracorporeal Membrane Oxygenation; ML: Machine Learning; NLP: Natural Language Processing; ATRs: Adverse Transfusion Reactions; CDSS: Clinical Decision Support System; EHR: Electronic Health Record; Hb: Hemoglobin; BMI: Body Mass Index; ICU: Intensive Care Unit; POD: Postoperative Delirium; DL: Deep Learning; VAP: Ventilator-Associated Pneumonia; AUC: Area Under the Curve; RBC: Red Blood Cell.

**Table 3:** Emerging recommendations (ER).

#	Recommendation	Description/Rationale	Representative References
1	Standardize and improve data quality	Interoperable, high-quality datasets are essential for accurate and generalizable AI models	[21,28,33,34]
2	Prioritize model interpretability	Clinicians require understandable AI outputs to trust and act on predictions	[20,21,30]
3	Validate models externally	Multicenter, prospective, or dynamic validation ensures reproducibility	[21,26,30,33]
4	Ensure ethical AI use	Address privacy, bias, and equity; employ privacy-preserving methods like federated learning	[19,27]
5	Integrate AI into clinical workflows	Training, infrastructure, and workflow adaptation are essential for effective implementation	[22,26,36]
6	Leverage AI for precision transfusion	Combine predictive analytics with omics data to optimize blood product quality and donor–recipient matching	[35,36]
7	Promote operational efficiency	Improve donor management, inventory control, and demand forecasting	[19,25,27,34]
8	Support real-time critical decision-making	AI augments rapid decisions in high-risk interventions, e.g., VA-ECMO and trauma care	[18,22,23,30]
9	Develop dynamic, time-dependent models	Time-aware models capture evolving patient states in ICU, trauma, and perioperative contexts	[28,30,33]
10	Encourage transparency and reproducibility	Open code, standardized reporting, and clear methodology foster trust and adoption	[21,28]

Abbreviations: AI: artificial intelligence; VA-ECMO: veno-arterial extracorporeal membrane oxygenation; ICU: intensive care unit.

**Table 4:** Market Segments and AI Integration Opportunities in Transfusion Medicine.

Segment	2024/2025 Value (USD Billion/Million)	Projected Value	CAGR (%)	AI Relevance/Opportunity
Global Transfusion Medicine Market	61.2 (2024)	88.8 (2030)	6.4	Broad AI adoption potential in diagnostics, decision support, supply forecasting
Transfusion Technology	14.4 (2023)	53.9 (2033)	14.11	Automation, AI-enabled processing, monitoring
Blood Transfusion Diagnostics	5.28 (2025)	8.79 (2034)	5.84	AI-assisted diagnostics, predictive testing
Blood Transfusion Diagnostics (alternate)	5.33 (2024)	9.64 (2032)	7.7	Same as above
Patient Blood Management (PBM)	14.65 (2024)	25.60 (2033)	6.40	AI-driven transfusion optimization, risk prediction
AI-driven Transfusion Decision Support	1.21 (2024)	6.38 (2033)	–	Real-time decision support, predictive analytics
AI-integrated Blood Analyzers	2805.7 million (2025)	19,348.6 million (2035)	21.3	Automated analysis, predictive quality control

Abbreviation: USD: US dollars.

## Data Availability

No new data were created or analyzed in this study.
